# Response to “Similarities in clinical course and outcome between juvenile idiopathic arthritis (JIA)-associated and ANA-positive idiopathic anterior uveitis: data from a population-based nationwide study in Germany”

**DOI:** 10.1186/s13075-023-03021-x

**Published:** 2023-03-14

**Authors:** Avery Zhou, Fatima Babiker, Andrew M. Philip, Stephen D. Anesi, C. Stephen Foster

**Affiliations:** 1 The Ocular Immunology and Uveitis Foundation, Waltham, MA USA; 2 Massachusetts Eye Research and Surgery Institution, 1440 Main St. Ste. 201, Waltham, MA 02451 USA; 3 Department of Ophthalmology, Harvard Medical School, Boston, MA USA

## Abstract

We have read the article entitled “Similarities in clinical course and outcome between juvenile idiopathic arthritis (JIA)-associated and ANA-positive idiopathic anterior uveitis: data from a population-based nationwide study in Germany” by Heiligenhaus et al. While we appreciate the work conducted by the authors, we have several comments we would like to address. First, the follow-up interval of 2 years is too short to conclude that the clinical course between two chronic pathologies is not significantly different. Second, remission status was determined by uveitis inactivity during the 2-year follow-up visit without any mention of flare frequency or length of remission, which is not a reliable measure of uveitis control. Third, ANA-positive idiopathic anterior uveitis is not a classification with a distinct clinical phenotype, and additional reports of serologic investigations would have been helpful.

Dear Editor,

We read, with great interest, the article by Heiligenhaus et al. [1]. While we appreciate the work conducted by the authors, especially population-based data on relatively rare conditions, we wish to raise a few points.

First, the study used a follow-up interval of 2 years to conclude that antinuclear antibody (ANA)-positive idiopathic uveitis and JIA-associated uveitis (JIA-U) do not significantly differ concerning the clinical course of uveitis, treatment, and response to corticosteroids and disease-modifying anti-rheumatic drugs (DMARDs). Although these pathologies may potentially behave similarly early in the disease course, this conclusion is potentially misleading because the clinical course of JIA-U is often much more chronic and stubborn than idiopathic anterior uveitis [2, 3]. Therefore, analysis over a longer period of time is necessary to more clearly elucidate differences in clinical course.

Second, remission status was determined by uveitis activity, or lack thereof, at the 2-year follow-up visit. While this is an important data point, this is an imprecise way to gauge remission status. With the current methodology, a patient who had been in remission for the entire 2 years and a patient with recurrent flares but quiet at the 2-year visit would both be considered in remission. Within the 2-year follow-up period, the number of flares or the duration of quiescence since the most recent flare would be important data, as these data more completely portray remission status.

Finally, ANA-positive idiopathic anterior uveitis is not a homogeneous classification with a distinct clinical phenotype. Among patients with idiopathic non-infectious anterior uveitis, ANA positivity is not known to produce a distinct clinical phenotype. The authors mention that human leukocyte antigen (HLA) B27 status was recorded, but there is no breakdown of its presence in the uveitis patients. HLA-B27-associated anterior uveitis has a known clinical phenotype, potentially adding to the heterogeneity of the ANA-positive idiopathic anterior uveitis group. Further presentation of serologic investigations would have been helpful.

We thank this group for their research on an under-represented topic in the literature and look forward to future work from this group.

## Data Availability

Not applicable.

## Abbreviations

ANA: Antinuclear antibody
DMARDs: Disease-modifying anti-rheumatic drugs
HLA: Human leukocyte antigen
JIA: Juvenile idiopathic arthritis
JIA-U: Juvenile idiopathic arthritis-associated uveitis
